# Path planning of scenic spots based on improved A* algorithm

**DOI:** 10.1038/s41598-022-05386-6

**Published:** 2022-01-25

**Authors:** Xingdong Wang, Haowei Zhang, Shuo Liu, Jialu Wang, Yuhua Wang, Donghui Shangguan

**Affiliations:** 1 College of Information Science and Engineering, Henan University of Technology, Zhengzhou, 450001 China; 2 School of Resources and Environmental Engineering, Anshun University, Anshun, 561000 China; 3 State Key Laboratory of Cryospheric Science, Northwest Institute of Eco-Environment and Resources, Chinese Academy Sciences, Lanzhou, 730000 China

**Keywords:** Computational science, Computer science

## Abstract

Traditional scenic route planning only considers the shortest path, which ignores the information of scenic road conditions. As the most effective direct search method to solve the shortest path in static road network, A* algorithm can plan the optimal scenic route by comprehensively evaluating the weights of each expanded node in the gridded scenic area. However, A* algorithm has the problem of traversing more nodes and ignoring the cost of road in the route planning. In order to bring better travel experience to the travelers, the above factors are taken into account. This paper presents a path planning method based on the improved A* algorithm. Firstly, the heuristic function of the A* algorithm is weighted by exponential decay to improve the calculation efficiency of the algorithm. Secondly, in order to increase the practicality of the A* algorithm, the impact factors that road conditions is introduced to the evaluation function. Finally, the feasibility of the improved A* algorithm is verified through simulation experiments. Experimental results show that the improved A* algorithm can effectively reduce the calculation time and road cost.

## Introduction

With the development of social economy, people's living standards have improved significantly, and tourism has become one of the most enthusiastic ways for people in their spare time. For tourists, the terrain of scenic spots is often rugged, so it is essential to keep enough energy to visit them. At the same time, for scenic spots, an efficient tour path can also reduce the congestion of scenic spots and improve the resource utilization of scenic spots, which is conducive to the sustainable and healthy development of scenic spots^1^. Therefore, reasonable tour path planning is very important to tourists' experience.

For the optimal path solving problem, researchers have proposed many classical algorithms including dijestra algorithm, Flow Direction algorithm^2^, etc. Dijkstra algorithm was proposed in 1959, and the algorithm is suitable for static networks, that is, when the weight in the network is fixed and there is no negative weight^3^. In the improvement of the algorithm, Zhang et al. proposed a path planning method based on dijkstra, which can save driving time and oil consumption^4^. Rosita et al. used vector normalization technology combined with dijkstra algorithm to achieve the optimal distribution path of products^5^. Sabri et al. used the combination of dijkstra algorithm and ant colony algorithm to find the safest escape path in high-rise buildings^6^. Ant colony algorithm was proposed by Italian scientist Dorigo according to the foraging process of animals, and the algorithm was originally used to solve the traveling salesman problem^7^. After that, many researchers improved the algorithm, for example, Zhou et al. optimized the intelligent logistics distribution path based on the improved ant colony algorithm, which was better to improve the dynamic optimization performance of the algorithm^8^. Yu et al. combined a special genetic operator in the ant colony algorithm, which not only avoids the local search limitations of the ant colony algorithm, but also enhances the global optimal searching ability of the ant colony algorithm^9^. In addition, there are many algorithms and improvements for solving the shortest path problem, such as, Miao et al. proposed an improved adaptive ant colony algorithm. While improving the real-time and security of robot path planning, balance the convergence and global search ability of ant colony algorithm, and transform the path planning problem into a multi-objective optimization problem by introducing multi-objective performance index, so as to realize the global comprehensive optimization of robot path planning^10^. Hsieh et al. proposed a route planning algorithm which combines the two-way fast-exploring random tree algorithm and greedy algorithm to generate various route planning schemes for ice navigation, and evaluated and selected a relatively optimal route with a lower risk scheme through a risk index^11^. Rakita et al. proposed a new sampling-based path planning method, which quickly finds solutions to high-dimensional path planning problems by minimizing the number of collision check samples^12^. Pan et al. used the improved floyd algorithm to design the optimal delivery path for take-out food, so that the travel time of the vehicle after optimization was shortened and the time efficiency was improved, but the algorithm did not consider the impact of road conditions^13^. Wu et al. combined normal distributed random numbers with genetic algorithms, and considered traveling least costs and the traveling highest experience index to construct the optimal tourism path^14^. Although the algorithms have been improved for their respective problems, some of their inherent shortcomings are difficult to eradicate. As the most effective direct search method for solving the shortest path in static road network, A* algorithm has been improved countless. Wang et al. Introduced the turning factor into the A* algorithm to solve the K shortest path problem. At the same time, they proposed a dynamic path planning method based on the A* algorithm, which can effectively search the shortest path and avoid collision^15^. Liu et al. Proposed an improved A* algorithm to solve the combination of normal channel and berthing channel^16^. Uttendorf et al. combined the fuzzy inference system with the A* algorithm to generate a path map for automatically guided vehicles^17^. Das et al. proposed an online path planning method based on an improved real-time A* algorithm, which plans the optimal path by avoiding obstructions and minimizing time, energy, and distance as the cost^18^. Shin et al. proposed an improved A* algorithm using Automatic Identification System (AIS) and weather data, and it finds the optimal paths by minimizing the estimated time of arrival generated by machine learning through 16-way node exploration^19^. Alani et al. proposed a new technique that consists of a hybridizing of A* algorithm and ant colony optimization, and the new technology can more accurately find the best parking path^20^. Pradhan et al. and Pardines both proposed to implement shopping guide path recommendations based on consumers' shopping lists, but they did not consider the problem of supermarket space modeling^21,22^. Ma et al. proposed a navigation path planning method for articulated underground scrapers based on improved A* algorithm to improve search efficiency^23^. Rahul et al. solved the problem of robotic path planning using a combination of A* algorithm and Fuzzy Inference, which finds the shortest path and generates the result in a finite time^24^.

In the above path planning study, there are many Dijkstra methods and swarm intelligence algorithms. Although the algorithms have been improved for their own problems, the inefficiency of the Dijkstra algorithm itself and the problem of the ant colony algorithm which is sensitive to the algorithm parameters are difficult to solve. In addition, the improvement of A* algorithm still has the problem of ignoring road cost. In order to provide better scenic route planning for passengers, this paper presents a route planning method based on improved A* algorithm. By exponentially weighting the heuristic function of the A* algorithm, the calculation efficiency of the algorithm is improved, and the A* algorithm is improved by using the road condition information of scenic spots as the evaluation index, which makes the algorithm more applicable to the actual scenic spot route planning.

## A* algorithm

The A* algorithm combines the advantages of the dijkstra algorithm and the breadth first search algorithm, which has better performance in the search^25,26^. The basic idea of the algorithm is as follows: First, find all neighbor nodes of the current search node. Second, compute the evaluation function value of each neighboring node, and the evaluation function in path planning is the distance from the initial node S to the goal node G. Finally, select the node which has the minimum evaluation function. The above process is repeated until the search reaches the end point.

The evaluation function of each node n is defined as follows:

1 $$ {\text{f}}({\text{n}}) = {\text{g}}\left( {\text{n}} \right) + {\text{h}}\left( {\text{n}} \right) $$

where f(n) denotes the estimated path cost from S to G, g(n) denotes the actual path cost from S to the current node, and h(n) is the heuristic function which denotes the estimated path cost from the current node to G. The heuristic function is generally expressed as Manhattan distance, Diagonal distance, or Euclidean distance.

The traditional A* algorithm has the problem that search nodes are too many and calculation time is too long. And heuristic function h(n) has an important influence on the speed and accuracy of path planning. Only by choosing a suitable h(n) value can we get the optimal path. To solve the problem, this paper improves the calculation efficiency of the A* algorithm by weighting the heuristic function. Besides, in order to increase the practicality, the impact factors (road conditions) are introduced to the evaluation function.

## Improvement of A* algorithm

### Exponential weighting in the heuristic function

In extreme cases, when the heuristic function h(n) = 0, the priority of nodes will be determined by g(n), then the algorithm degenerates to dijkstra algorithm. If h(n) is always less than or equal to the cost from the current node to G, then A* algorithm guarantees that it can find the shortest path. But the smaller h(n) is, the more A* algorithm will traverse nodes, which will lead to the slower search. Therefore, the weighting of h(n) in the heuristic function should be increased. The exponential attenuation method was proposed for weighting by Wang et al. as shown in Eq. (2), and the improved search efficiency is further improved^27^.

2 $$ {\text{f}}({\text{n}}) = {\text{g}}\left( {\text{n}} \right) + \exp \left[ {{\text{h}}\left( {\text{n}} \right)} \right]\;*\;\left[ {{\text{h}}\left( {\text{n}} \right) + {\text{h}}\left( {\text{p}} \right)} \right] $$

where h(p) is the distance from the parent node of current node to the target node. exp[h(n)] is the weighting of the heuristic function.

### Improvement of valuation function

It not only needs to quickly plan paths for tourists, but also needs to consider the road ups and downs in scenic areas to provide the best route for tourists to save time and effort. In this paper, the path planning for scenic spots is improved on the basis of Eq. (2). And the valuation function of A* algorithm needs to be modified accordingly. g(n) in the traditional A* algorithm is the total distance from the starting node S to the current node n. This paper introduces one impact factors (road condition level p(n)) to the evaluation function, The improved g(n) is as follows:

3 $$ {\text{g}}({\text{n}}) = \sum\limits_{{{\text{i}} = 1}}^{{\text{m}}} {{\text{p}}({\text{n}})} $$

where m represents the number of road sections passed from the starting node S to the current node n. p(n) can be set as linear functions f(x) = ax + b, where x represents the actual distance of the road^28^. For terrain with different road conditions, the terrain undulation can be set to r, and a can be calculated according to the calculation function (The calculation function is a = c*r + d). This paper set r = 30°, 45°, 60°; c = 0.2, d = 1, b = 1, and we can get Eq. (4) as follows:

4 $$ {\text{f}}_{{\text{p}}} {\text{(x) = (0}}{\text{.2r + 1)x + 1}} $$

From the above Equation (3), (4), we can obtain the Equation (5) as follows

5 $$ {\text{g}}({\text{n}}) = \sum\limits_{{{\text{i}} = 1}}^{{\text{m}}} {\left( {0.2{\text{r}} + 1} \right){\text{x}} + 1} $$

The heuristic function h(n) can be set by exponential weighting according to Eq. (3) and Eq. (6). The evaluation function of the improved A* algorithm can be expressed as follows:

6 $$ {\text{f}}({\text{n}}) = \sum\limits_{{{\text{i}} = 1}}^{{\text{m}}} {\left( {0.2{\text{r}} + 1} \right){\text{x}} + 1} + \exp \left[ {{\text{h}}\left( {\text{n}} \right)} \right]\;*\;\left[ {{\text{h}}\left( {\text{n}} \right) + {\text{h}}\left( {\text{p}} \right)} \right] $$

**Figure 1:**
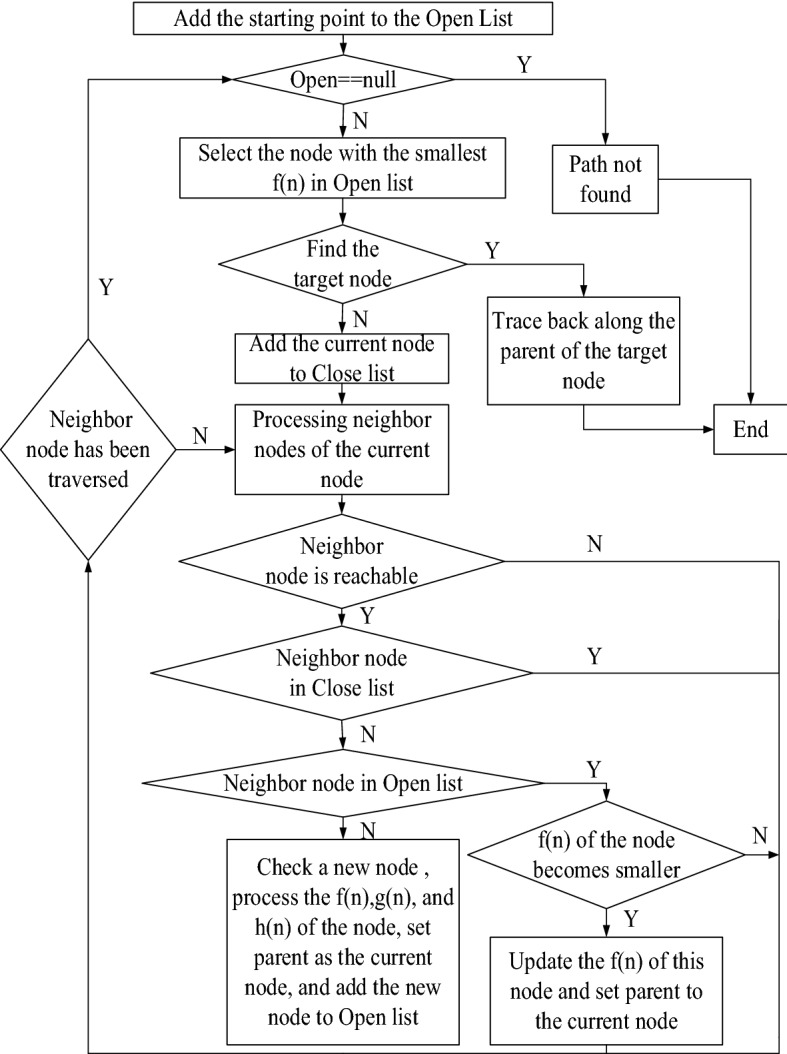
Flow chart of improved A* algorithm.

This paper uses two lists (Open list and Close list) to store the relevant node information. The Open list mainly saves the node information that has been generated but has not been accessed, while the Close list mainly saves the visited node information. The fake code for he improved A* algorithm is listed as follows:

The flow chart of the improved A* algorithm is shown in Fig. 1.

## Results

### Process of path planning

**Figure 2:**
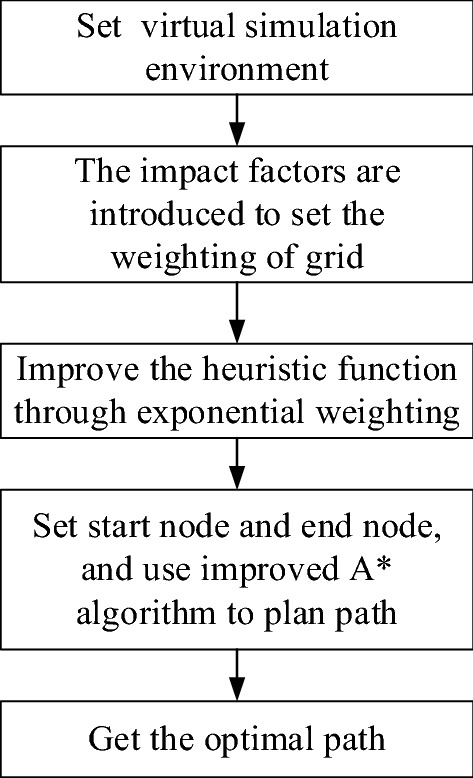
Flow chart of path planning.

First, introduce the road condition level to construct a dynamic road network data model for scenic spots. Then, the heuristic function is improved by exponential weighting. Finally, the start and end points of path planning are determined, and the improved A* algorithm is used for path planning. The path planning process is shown in Fig. 2:

### Simulation experiments

**Figure 3:**
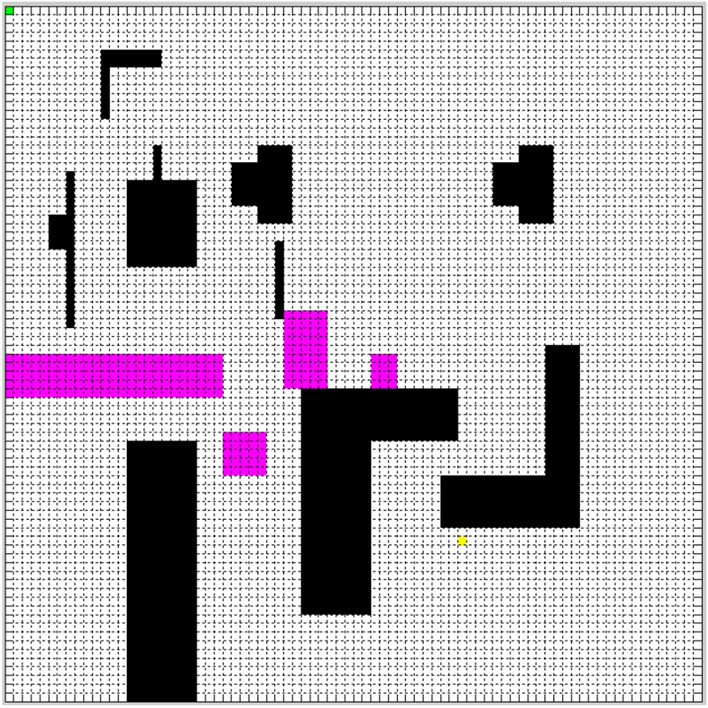
Simulation map.

The side length of the square is 80 in Fig. 3, and it is divided into 80 × 80 grids. The simulation experiment of the path planning is done by MATLAB R2010b shown in Fig. 3 in the simulation map. Take the top left corner of the simulation map as the origin of the coordinates, and the start point is set to (1, 1) represented by a green point, and the end point is set to (62, 53) represented by a yellow point. Pink polygons indicate that road undulation angle is larger than 30°, and black polygons represent obstacles.

**Figure 4:**
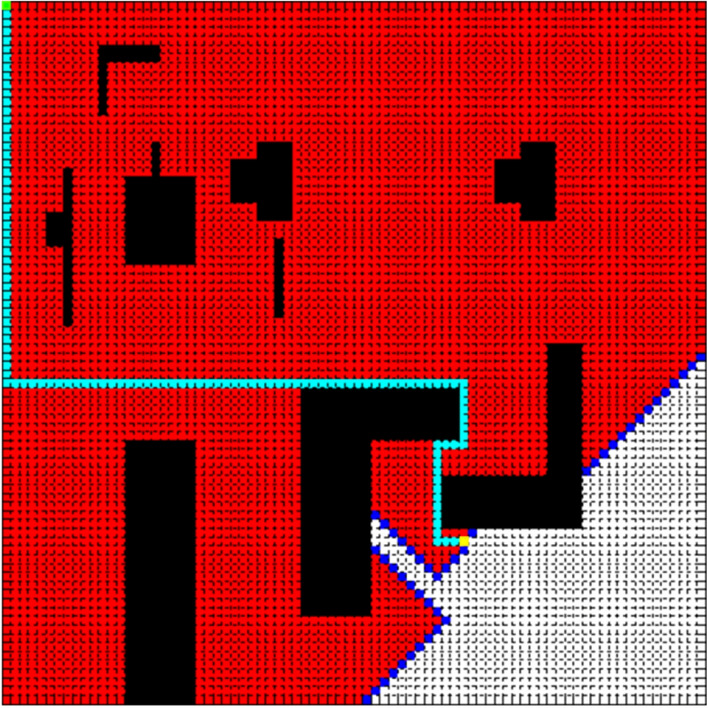
Dijkstra algorithm.

**Figure 5:**
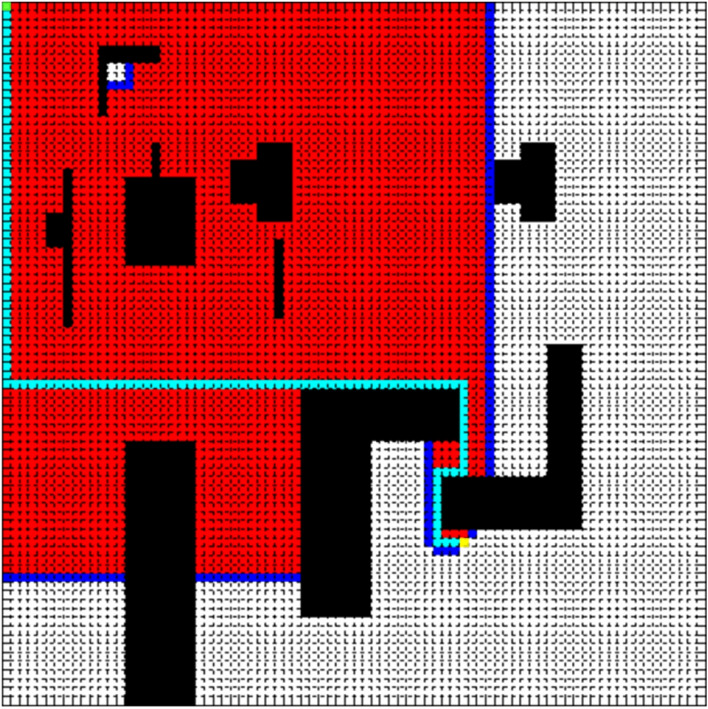
A* algorithm.

**Figure 6:**
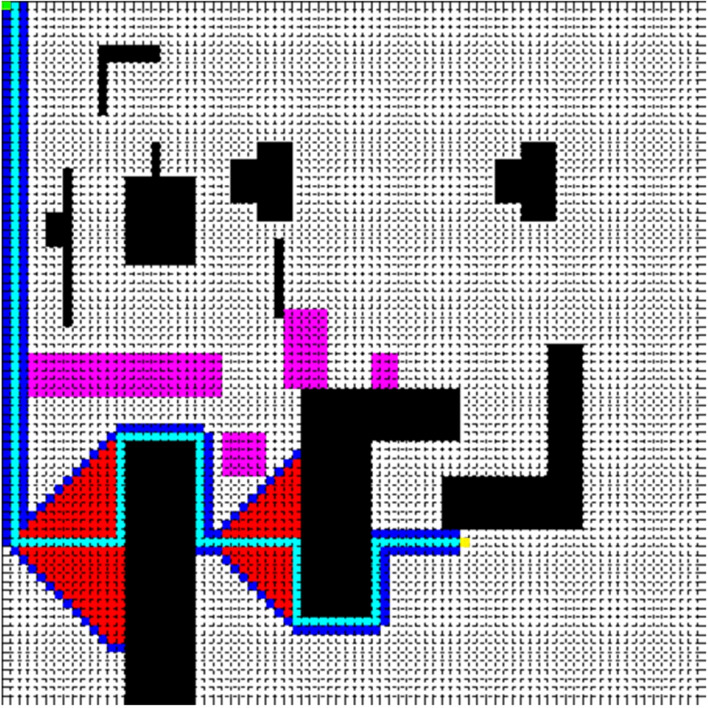
Improved A* algorithm.

In order to verify the feasibility of the improved A* algorithm, we add dijkstra algorithm, A* algorithm for comparison and evaluate the optimization effect from three aspects: Number Of Nodes Traversed (NONT), Path Cost (PC), and Path Length (PL). Figures 4, 5 and 6 compare the effect of path planning, and Table 1 is the parameters comparison.

**Table 1:** Parameter comparision.

Algorithm	NONT	PC	PL
Dijkstra	4631	135	119
A*	2871	135	119
Improved A*	380	94	155

In Figs. 4, 5 and 6, the red area is the node area to be traversed by the algorithm, and the blue is the boundary of the traversed area, and the cyan is the path, and the purple is the area with a slope greater than 30.

The results presented in Table 1 shows that the improved A* algorithm can effectively reduce the calculation time and road cost. Compared to the traditional A* algorithm, although the improved A* algorithm increased the path length by 30.2%, it reduced the number of nodes traversed and path cost by 86.8% and 30.3% respectively. Compared to the dijkstra algorithm, it reduced the number of nodes traversed and path cost by 91.8% and 30.3% respectively.

## Discussion and conclusion

In order to solve the problem that the A* algorithm ignores the road cost in path planning, this paper proposes a path planning method based on improved A* algorithm. Firstly, the heuristic function of the A* algorithm is weighted to improve the calculation efficiency. Secondly, in order to increase the practicality, the impact factors (road conditions) is introduced to the evaluation function. The feasibility of the improved A* algorithm is verified by comparing with dijkstra algorithm and traditional A* algorithms. Experimental results show that the improved A* algorithm can effectively reduce the calculation time and road cost.

Compared to the traditional A* algorithm and dijkstra algorithm, the improved A* algorithm achieves remarkable results, and it is more suitable for the path planning of Scenic spots. It can be seen that dijkstra algorithm and traditional A* algorithm both traverse a lot of nodes and ignore the road cost on finding the optimal path. Besides, as can be clearly seen from the Table 1, the number of traversed nodes decreases from 4631 to 381 nodes, on the other hand, the path cost decreases from 115 to 94. The improved A* algorithm is slightly stronger in real time and practicality.

In the simulation experiments, we assume that the environment is completely known, that is, obstacles, road conditions, start point and end point are custom-designed, so the simulation environment can't represent all the scenic environment. Accordingly, path planning research in unknown environments may be the next step of our research.

Moreover, impact factors, such as road conditions and the popularity of scenic spots, have not been statistically examined at present due to the difficulty in measuring and establishing the simulation environment of scenic spots. Terrain undulation is set to 30°, 45°, 60° and the popularity of scenic spots is set to 1, 1.5, 2 in this paper. However, there are a great many factors influencing path planning, and we only selectively discussed some common factors due to the restriction of data. Other factors, such as path's congestion, tourism emergency, move speed of tourists, are not included. This will be a focus of future research, and it should take different mode of dividing according to different scenic spots.

Compared to the dijkstra algorithm and traditional A* algorithm, although the improved A* algorithm reduced the number of nodes traversed and path cost effectively, the path length increased from 119 to 155. Therefore, it is suggested that the future work should be focused on reducing the path length of improved A* algorithm.

## Data Availability

The data that support the findings of this study are available from the corresponding author upon reasonable request.
